# Identification of five novel mutations in the long isoform of the *USH2A* gene in Chinese families with Usher syndrome type II

**Published:** 2008-11-17

**Authors:** Hanjun Dai, Xiaohui Zhang, Xin Zhao, Ting Deng, Bing Dong, Jingzhao Wang, Yang Li

**Affiliations:** Beijing Institute of Ophthalmology, Beijing Tongren Hospital, Capital Medical University, Beijing, China

## Abstract

**Purpose:**

Usher syndrome type II (USH2) is the most common form of Usher syndrome, an autosomal recessive disorder characterized by moderate to severe hearing loss, postpuberal onset of retinitis pigmentosa (RP), and normal vestibular function. Mutations in the *USH2A* gene have been shown to be responsible for most cases of USH2. To further elucidate the role of *USH2A* in USH2, mutation screening was undertaken in three Chinese families with USH2.

**Methods:**

Three unrelated Chinese families, consisting of six patients and 10 unaffected relatives, were examined clinically, and 100 normal Chinese individuals served as controls. Genomic DNA was extracted from the venous blood of all participants. The coding region (exons 2–72), including the intron-exon boundary of *USH2A*, was amplified by polymerase chain reaction (PCR). The PCR products amplified from the three probands were analyzed using direct sequencing to screen sequence variants. Whenever substitutions were identified in a patient, restriction fragment length polymorphism analysis, or single strand conformation polymorphism analysis was performed on all available family members and the control group.

**Results:**

Fundus examination revealed typical fundus features of RP, including narrowing of the vessels, bone-speckle pigmentation, and waxy optic discs. The ERG wave amplitudes of three probands were undetectable. Audiometric tests indicated moderate to severe sensorineural hearing impairment. Vestibular function was normal. Five novel mutations (one small insertion, one small deletion, one nonsense, one missense, and one splice site) were detected in three families after sequence analysis of *USH2A*. Of the five mutations, four were located in exons 22–72, specific to the long isoform of *USH2A*.

**Conclusions:**

The mutations found in our study broaden the spectrum of *USH2A* mutations. Our results further indicate that the long isoform of *USH2A* may harbor even more mutations of the *USH2A* gene.

## Introduction

Usher syndrome (USH) is an autosomal recessive disorder characterized by retinitis pigmentosa (RP) and sensorineural hearing impairment, with or without vestibular dysfunction. It is a clinically and genetically heterogeneous condition. Clinically, USH is subdivided into three types: USH type I (USH1), USH type II (USH2), and USH type III (USH3). USH1 is the most severe form of this disease characterized by congenital profound hearing loss, prepuberal onset of RP, and vestibular dysfunction. Patients with USH2 experience congenital moderate to severe hearing impairment and postpuberal onset of RP with intact vestibular function. Patients with USH3 show progressive postlingual hearing loss, later onset of RP, and variable vestibular dysfunctions. Of the three clinical types, USH2 is the most common form of USH, and it may account for more than half of the patients with USH [1,2]. To date at least 11 chromosome loci have been mapped for the three clinical USH types. Three of them are assigned to USH2: *USH2A* (*USH2A*), *USH2C* (*VLGRI*), and *USH2D* (*DFNB31*) [1,3-5].

The *USH2A* gene located on chromosome 1q41 has two alternatively spliced isoforms: a short *USH2A* isoform a and a much longer *USH2A* isoform b. In 1998, *USH2A* isoform a was first identified and found to contain 21 exons encoding a protein of 1,546 amino acids [6]. However, in 2004, van Wijk et al. [7] identified 51 additional exons at the 3′ end of *USH2A*. The longer *USH2A* alternative splicing isoform b, comprised of 72 exons, encodes a putative protein of 5,202 amino acids. USH2A isoform a is thought to be an extracellular matrix protein involved in protein–protein or protein-matrix interactions, while USH2A isoform b is a transmembrane protein [3,8]. In the inner ears of mice and rats, USH2A isoform b is a component of the ankle links that connect the differentiating stereocilia of the hair cells [9]. In the photoreceptor, it is an essential partner of a protein network that is likely to be involved in cargo delivery from the inner segment to the outer segment [10]. *USH2A* is thought to be involved in 74 to 90 percent of cases of USH2 [11]. Moreover, mutations of *USH2A* could also be responsible for atypical USH and nonsyndromic RP [11-13].

In this study, we performed a mutation screening of *USH2A* corresponding to *USH2A* isoform b. The screening was done in three Chinese families who were affected with USH2, and we identified five novel mutations.

## Methods

### Clinical data and sample collection

This study was granted approval by the Beijing Tongren Hospital Joint Committee on Clinical Investigation and conformed to the tenets of the Declaration of Helsinki. Three Chinese families with USH2 were referred to Beijing Tongren Hospital. After informed consent was obtained from each family member, all participants underwent careful clinical examinations, including best-corrected visual acuity, slit-lamp, and ophthalmoscope. Three probands of the families were given electroretinograms and they also received audiometric and vestibular tests. Full field electroretinogram were performed according to International Society for Clinical Electrophysiology of Vision (ISCEV) protocols. USH2 was diagnosed based on the clinical history, typical RP fundus appearance, sensorineural hearing impairment, and intact vestibular function.

### Mutation detection

Blood samples of all participants were obtained by venipuncture and genomic DNA was extracted by standard phenol protocols. Mutation screening was performed in the three families using direct DNA sequence analysis. The coding region (exons 2–72) of *USH2A* was amplified by polymerase chain reaction (PCR) in the probands of the three families. The pairs of primers for exons 2–21 were used as designed by Weston et al. [11], except those for exons 6 and 7. Those for exons 22–72 were used as described by van Wijk et al. [7], except those for exons 41, 53, 63, 64, and 71 (Appendix 1). For direct sequencing, PCR products were purified (Shenneng Bocai PCR purification kit; Shenneng, Shanghai, China). The purified PCR products were sequenced using an automatic fluorescence DNA sequencer (ABI, Prism 373A; Perkin Elmer, Foster City, CA), according to the manufacturer’s instructions. Nucleotide sequences were compared with the published cDNA sequence of *USH2A* (GenBank NM_206933.1). For *USH2A* gene cDNA numbering +1 corresponds to A in the ATG translation initiation codon in RefSeq (AY481573.1).

### Restriction fragment length polymorphism (RFLP) analysis

Variations found in the sequencing were confirmed with the restriction endonucleases Taq^a^I, MslI, and EcoRV (New England Biolabs, Ipswich MA), which were used with all available family members and 100 normal controls. The reaction was performed in a 10 µl volume containing 9.4 µl PCR product, 0.1 μl BSA (100 μg/ml), and 0.5 μl enzyme (4 U/μl). The reaction was incubated overnight at 65 °C for Taq^a^I and 37 °C for MslI and EcoRV. Afterwards the whole digest was run on a 1% agarose gel and visualized under ultraviolet light.

### Single strand conformation polymorphism

For the sequence variants that did not change (create or erase) a restriction site, single strand conformation polymorphism (SSCP) was used with all available family members and the control group. As the PCR fragments used in SSCP analysis were between 150 to 300 bp, two pairs of specific primers were designed for detecting the mutation in exons 43 and 61 (Appendix 1). Amplified DNA was mixed with an equal volume of formamide buffer: 95% formamide, 10 mM EDTA, 0.1% bromophenol blue, and 0.1% xylene cyanol. Denatured samples were electrophoresed on a 16% nondenaturing polyacrylamide gel, with an acrylamide:bisacrylamide ratio of 49:1, for 12–16 h at 300 v and 4 °C, before gels were silver-stained and analyzed.

### Prediction of splice scores

A Splice View program was used to assess the possibility that intron sequence variants could create or exclude splice sites. The program is accessible at splice.

## Results

**Figure 1:**
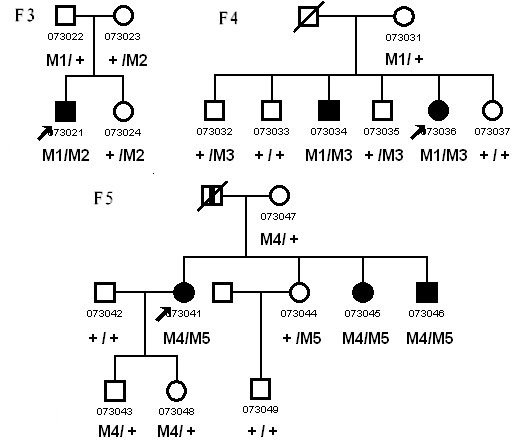
Pedigree of the three Chinese families with Usher syndrome type II who had mutations in *USH2A*. Males and females are represented by squares and circles, respectively. The symbols for affected family member are filled. The symbols for deceased family members have a slash. The symbols for proband have an arrow. The genotype of each evaluated individual is shown below the individuals’ symbol and identification number. Abbreviations: Wild type (+); c.8559-2A>G (M1); p.T3936P (M2); p.R34fs (M3); p.S2828fs (M4); p.W3150X (M5).

**Figure 2:**
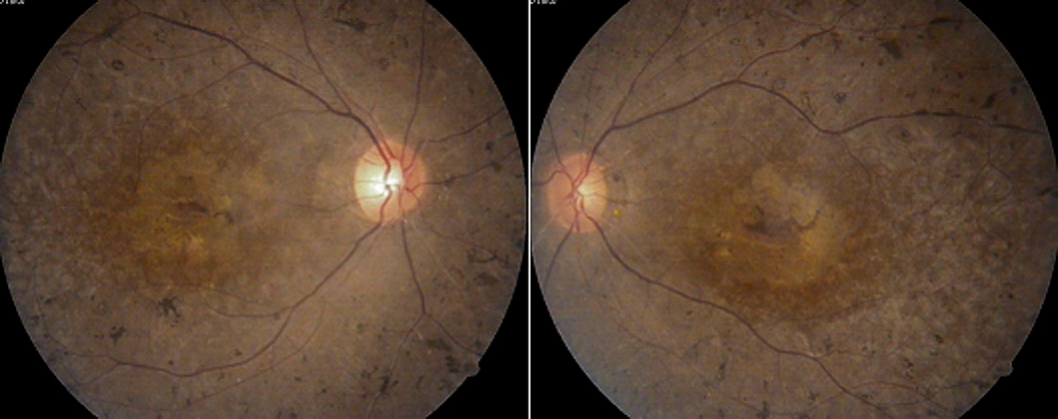
Fundus appearance of a patient with Usher syndrome type II. Fundal photographs from a proband (family F5) show typical retinal degeneration with attenuation of the retinal vessels, irregular pigment clumps in the retina, and waxy pallor of the optic nerve head.

We have identified three unrelated Chinese families consisting of six patients diagnosed with USH2 and 10 unaffected relatives. The inheritance pattern in these three families was autosomal recessive (Figure 1). All of the patients experienced night blindness, vision acuity impairment, and moderate to severe hearing loss. Ophthalmoscopic examination demonstrated attenuation of the retinal vessels, bone-speckle pigmentation in the midperiphery of the fundus, and waxy pallor of the optic nerve head (Figure 2). The ERG wave amplitudes of three probands were indistinguishable from baseline (data not shown). Audiometric tests indicated moderate to severe sensorineural hearing impairment. Vestibular function was normal. Detailed clinical information on affected family members is summarized in Table 1.

**Table 1:** Clinical features of the patients from the three Chinese families with Usher syndrome type II.

**Family number**	**Patient**	**Best corrected visual acuity (R/L)**	**Onset age of night blindness (year)**	**Fundus appearance**	**Onset age of hearing loss (year)**	**Hearing impairment**	**ERG**	**Vestibular function**
F3	73021	FC/PL	14	RP	10	Moderate (SP)	Wave undetectable	Normal
F4	73034	0.3/0.2	15	RP	congenital	Moderate (SP)	N/A	Normal
	73036	FC/ PL	14	RP	congenital	Severe (SP)	Wave undetectable	Normal
F5	73041	HM/HM	14	RP	congenital	Severe (SP)	Wave undetectable	Normal
	73045	HM/HM	10	RP	congenital	Severe (SP)	N/A	Normal
	73046	0.1/0.1	14	RP	congenital	Moderate (SP)	N/A	Normal

Abbreviations: R, right eye; L, left eye; FC, finger counting; PL, perception of light; HM, hand motion; SP, slight progressive; N/A, data not available.

**Figure 3:**
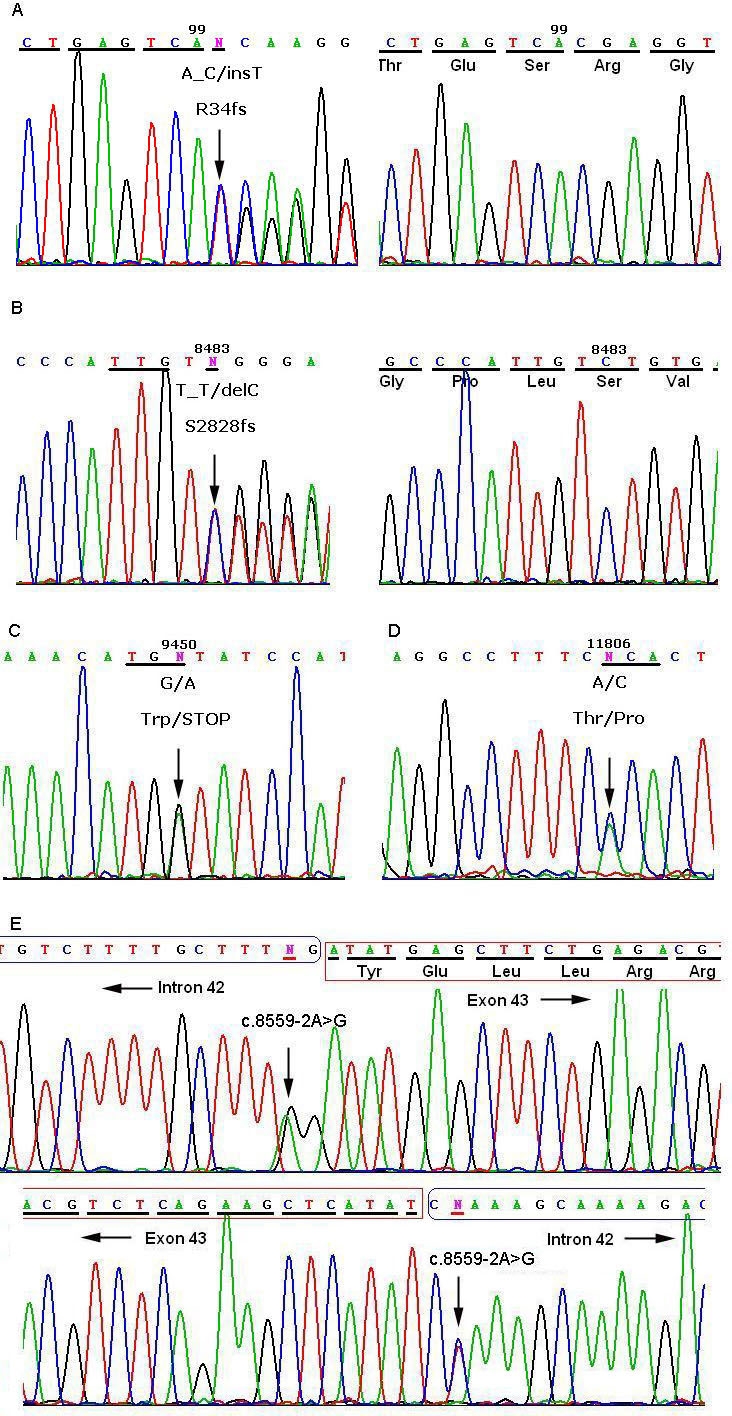
Direct sequencing analysis of the coding region of the *USH2A* gene. **A:** Left, heterozygous one base insertion mutation c.99_100insT (p.R34fs) detected in patient 073036; right, the corresponding wild-type sequence. **B**: left, Sequence shows the heterozygous one base deletion mutation c.8483delC (p.S2828fs) identified in patient 073041 and right, the corresponding wild type sequence. **C**: Sequence presents the heterozygous nonsense mutation c.9450G>A (p.W3150X) detected in patient 073041. **D**: Sequence shows the heterozygous missense mutation c.11806A>C (p.T3936P) identified in patient 073021. **E**: upper, Sequence presents the heterozygous splicing site mutation c.8559-2A>G identified in patient 073021, lower, the corresponding complimentary strand sequence.

**Figure 4:**
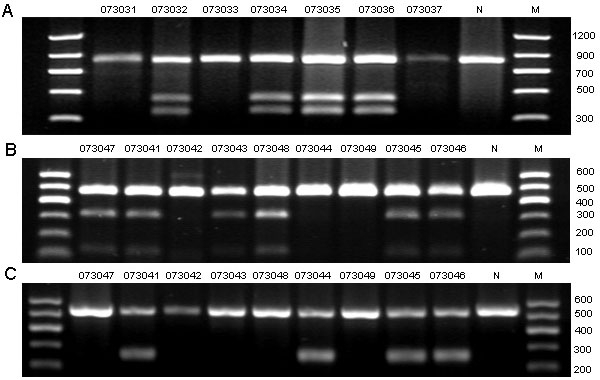
Restriction fragment length analysis on the three mutations detected in this study. **A**: c.99_100insT created a Taq^a^I restriction site that cosegregated with the affected individuals and the carriers (370 bp, 470 bp, and 840 bp), but not with unaffected individuals and normal controls (840bp). **B:** c.8483delC created an MslI restriction site that cosegregated with the affected individuals and the carriers (150 bp, 340 bp, and 490 bp), but not with unaffected individuals and normal control (490 bp). **C**: c.9450G>A created an EcoRV restriction site that cosegregated with the affected individuals and the carriers (265 bp, and 530 bp), but not with unaffected individuals and normal control (530 bp). Participant identification number is given above each lane. N represents the normal control, and M refers to the DNA ladder in bp.

**Figure 5:**
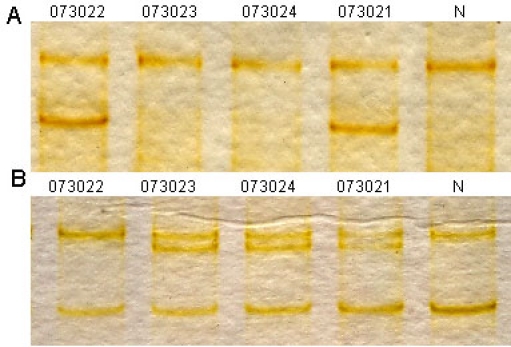
Single strand conformation polymorphism analysis for the two mutations identified in the current study. **A:** Single strand conformation polymorphism (SSCP) analysis for the heterozygous mutation c. 8559–2A>G revealed that the mutant pattern (two bands) cosegregated with the affected individuals and carriers, but not with the unaffected individuals and normal control (one band). **B:** SSCP analysis for c.11806A>C (p.T3936P) showed that the mutant pattern (three bands) cosegregated with the affected individuals and carriers, but not with the unaffected individuals and normal control (two bands). Participant identification number is listed above each lane. N represents the normal control.

Sequencing of *USH2A* revealed 17 sequence variants in this study, five of which were predicted to be pathogenic mutations (Table 2). All five disease-causing mutations were heterozygous and were first detected in our study (Figure 3). Mutation c.8559–2A>G was identified in two families, and the other four mutations (p.T3936P, p.R34fs, p.S2828fs, and p.W3150X) were only detected in one patient or in in one family. Using RFLP or SSCP analysis, we confirmed the five mutations cosegregated with the USH2 phenotype (Figure 1, Figure 4, and Figure 5), but we did not detect them in 100 normal controls.

**Table 2:** Novel mutations in *USH2A* identified in this study

**DNA change**	**Exon**	**Protein change**	**Type of nucleotide change**	**Family number**	**Frequency**
c.99_100insT	2	p.R34fs	Heterozygous	F4	0/200
c.8483delC	42	p.S2828fs	Heterozygous	F5	0/200
c.8559–2A>G	43	Exon43DEL	Heterozygous	F3,F4	0/200
c.9450G>A	48	p.W3150X	Heterozygous	F5	0/200
c.11806A>C	61	p.T3936P	Heterozygous	F3	0/200

The "Frequency" column, shows the number of chromosomes.

Three different combinations of heterozygous mutations were observed in the three families. In families F3 and F4, one allele harbored the same splice site mutation c.8559–2A>G; the other allele carried a missense mutation c.11806A>C (p.T3936P) and a small insert mutation c.99_100insT (p.R34fs), respectively. Splice site prediction software online (splice) was used, and c.8559–2A>G was predicted to abolish the splice accept site of exon 43, which would lead to the skipping of exon 43. In family F5, one allele harbored a deletion mutation c.8483delC (p. S2828fs), while the other allele carried a nonsense mutation c.9450G>A (p.W3150X).

In addition to the five pathogenic mutations detected in this study, 12 nonpathogenic sequence variants were identified. Four of these were novel, including two silent variants (c.879T>G/p.=and c.1935A>T/p.=), one intron variant (c.10388–27T>C), and one missense variant (c.9340C>T/p.P3114S). Analysis by the online splice prediction software indicated that variant c.10388–27T>C did not disrupt any splice donor site or accept site, and it didn’t create a new splice site. Although p.P3114S was detected in family F5 and cosegregated in family members with the disease in the family, it occurred both in the nonfunction domain and nonconserved region of USH2A isoform b. Furthermore, it was also identified in two individuals among the 100 normal controls. It was classified as a nonpathogenic variant. Table 3 summarizes these variants on the basis of their nature and frequency.

**Table 3:** Presumed nonpathogenic variants found in *USH2A*.

**Exon**	**Nucleotide change**	**Codon change**	**Family** **number**	**Allele frequency**	**Source**
6	c.879T>G	p.L293L	F5	N/A	This study
11	c.1935A>T	p.T645T	F5	N/A	This study
18	IVS17–8T>G		F5	N/A	[11]
21	c.4457A>G	p.K1486R	F5	76/180	[11]
34	c.6506T>C	p.I2169T	F3,F4,F5	27/100	[16]
47	c.9340C>T	p.P3114S	F5	2/200	This study
52	c.10232A>C	p.E3411A	F3,F4,F5	23/64*	[17]
53	IVS52–27T>C		F3,F4,F5	N/A	This study
60	c.11602A>G	p.M3868V	F5	8/64*	[17]
63	c.12612A>G	p.T4204T	F3,F4,F5	N/A	[17]
	c.12666A>G	p.T4222T	F5	14/64*	[17]
	c.13191G>A	p.E4397E	F3,F4,F5	7/64*	[17]

Abbreviations: N/A, data not available; the asterisk indicates that the allele frequency referred to patients sample.

## Discussion

In this study, we examined three Chinese families with clinically diagnosed USH and screened the *USH2A* gene. We identified five novel mutations in the three families, which included three point mutations, one insertion mutation, and one deletion mutation. As shown in Figure 1, the three families carried different compound heterozygous mutations, which were consistent with a pattern of autosomal recessive inheritance of USH. Although all affected individuals experienced progressive hearing loss, the progression was slight, which was not as evident as it was in USH3. The clinical phenotype could be considered to be USH2.

**Figure 6:**
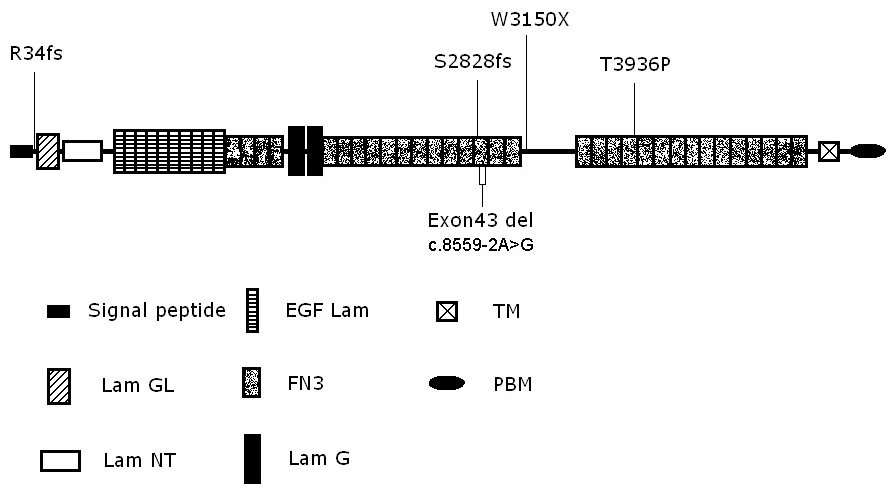
Schematic distribution shows the five novel mutations identified in the current study along the USH2A isoform b protein domains. Abbreviation: EGF-like domain (EGF Lam);  Transmembrane region (TM); Laminin G-like domain (Lam GL); Fibronectin type III (FN3); PDZ-binding motif (PBM); Laminin N-terminal (Lam NT); Laminin G domains (Lam G).

As shown in Figure 6, USH2A isoform b consists of eight domains: N-terminal signal peptide, laminin G-like domain (Lam GL), laminin N-terminal (Lam NT), laminin-type EGF-like domain (EGF Lam), fibronectin type III (FN3), laminin G domains (LamG), transmembrane region (TM), and cytoplasmic domain with a PDZ-binding motif at its C-terminal end [7]. Four novel truncating mutations (p.R34fs, p.S2828fs, p.W3150X, and c.8559–2A>G), either directly or indirectly result in premature termination of USH2A translation or lead to deletion of more than one amino acid residue. This may cause significant changes in the primary protein structure. The c.99_100insT results in a frameshift at codon 34 and premature stop codon 8 amino acids downstream, which removes almost all protein except for the N-terminal signal peptide. The c.8483delC, located in the 14th FN3 repeat, causes a frameshift at codon 2828 and a premature stop codon 1 amino acid downstream. The nonsense mutation c.9450G>A leads to a premature stop codon at 3150. As the three premature stop codons are not located in the last exon of *USH2A*, there is a possibility that nonsense-mediated mRNA decay might be involved in abnormal RNA processing. Nonsense-mediated mRNA decay is a widespread cellular process that proofreads nascent mRNA transcripts and destroys those that bear premature termination codons before they are actually translated into truncated and potentially harmful proteins [14]. The splicing variant c.8559–2 A>G may result in the skipping of exon 43 of *USH2A*. The skipping of exon 43 doesn’t result in the interruption of the reading frame. However, it results in the deletion of 41 amino acids, which encode most regions of the 15th FN3 repeat.

**Figure 7:**
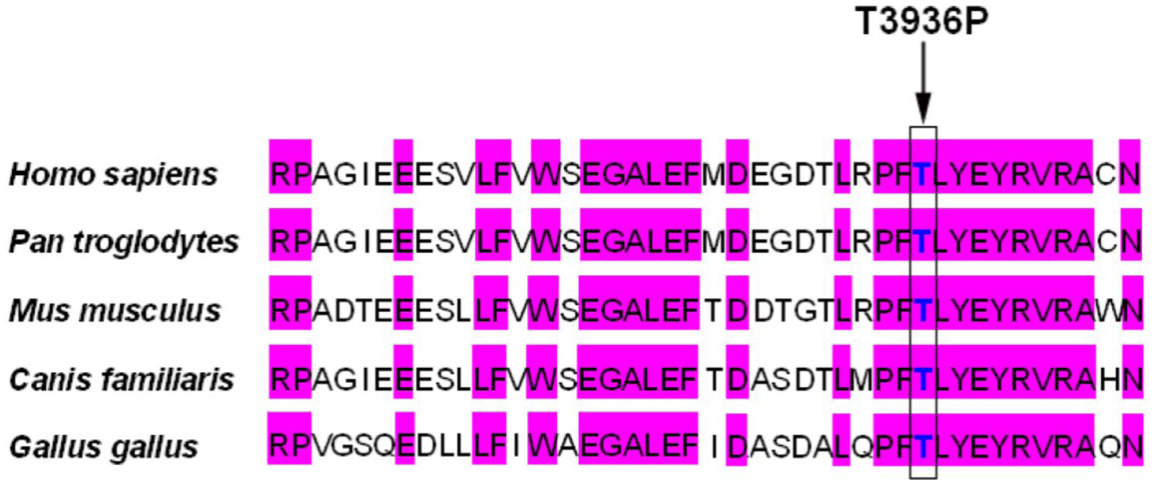
Sequence alignment portion of the 21 FN3 domain spanning the novel missense mutation p.T3936P of human USH2A isoform b with other species. Identical residues are highlighted in purple. Arrow marks the identified mutation.

Unlike the mutations that cause the loss of a large part of the protein, most of the amino acid substitutions occur at highly conserved sites and result in significant change in polarity or charge. The p.T3936 is located in the 21 FN3 repeat and is highly conserved in humans, mice, chicks, and dogs. The only missense mutation of p.T3936P (c.11806A>C) replaces a polar threonine with a nonpolar hydrophobic proline (Figure 7).

To date, more than 160 mutations have been reported in *USH2A*; however, mutation screening for Chinese patients was performed only once (the *USH2A* gene; Universal Mutation Database, UMD). Liu et al. [12] only screened the c.2299delG mutation in five Chinese families with USH2 and atypical USH and detected one homozygous and one heterozygous c.2299delG. This is the first report to describe mutation analysis of the complete coding sequence of *USH2A* in the Chinese population. The c.2299delG was the most common mutation with an allele frequency of 14–50% in patients from Europe and North America [6,11-13,15-19]. Dreyer et al. [20] found that c.2299delG was the result of an ancestral mutation that had spread throughout Europe and into the New World as a result of migration. The c.2299delG was only detected in single patients from South America, South Africa, and China [12,20]. It was not identified in the current study. As we only underwent mutation screening for three families, it is difficult to reach the conclusion that c.2299delG is a low frequency allele in the Chinese patients.

Of the five novel mutations detected in the current study, four are located in exons 22–72, which are specific to the long isoform of USH2A. Two families (F3 and F5) carrying compound heterozygous mutations (either two truncating or one truncating combined with one missense) in exons 22–72, presented with the USH2 phenotype. This is in accordance with Dreyer’s observation in a large unrelated Scandinavian patients study [21].

In conclusion, we have described five novel mutations in three Chinese families with USH2. Our findings expand the spectrum of *UHS2A* mutations in USH and disclose that the long isoform of USH2A may harbor even more mutations of the *USH2A* gene.

## Appendix 1. PCR primers used in this study

Oligonucleotide primers used for PCR amplification and sequencing of *USH2A*. To access the data, click or select the words “Appendix 1.” This will initiate the download of a pdf archive that contains the file.
